# Participatory Action Research to Enhance Patient‐Centred Goal Setting in Geriatric Rehabilitation: A Nursing Team's Quest

**DOI:** 10.1111/jan.16774

**Published:** 2025-01-24

**Authors:** Anne Marie Vaalburg, Elizabeth M. Wattel, Petra Boersma, Cees M. P. M. Hertogh, Robbert J. J. Gobbens

**Affiliations:** ^1^ Department of General Practice and Elderly Care Medicine, Amsterdam Public Health Research Institute, Amsterdam UMC Vrije Universiteit Amsterdam Amsterdam The Netherlands; ^2^ Faculty of Health, Sports and Social Work Inholland University of Applied Sciences Amsterdam The Netherlands; ^3^ Ben Sajet Center Knowledge Center for Long‐Term Care Amsterdam The Netherlands; ^4^ Department of Primary and Interdisciplinary Care, Faculty of Medicine and Health Sciences University of Antwerp Antwerp Belgium; ^5^ Tranzo, Tilburg School of Social and Behavioral Sciences, Tilburg University Tilburg The Netherlands

**Keywords:** action research, geriatric rehabilitation, nursing role, older people, patient engagement, patient involvement, patient‐centred care

## Abstract

**Aim:**

The aim of this study was to provide insights into how, through exploring goal‐setting interventions, a nursing team in geriatric rehabilitation might refine their patient‐centred strategies.

**Design:**

The study design was participatory action research (PAR).

**Method:**

Team members and nursing students, under the guidance of a facilitator, performed two PAR cycles. In the first cycle, the action phase consisted of preparing a multidisciplinary team meeting (MTM) with a patient. In the second cycle, based on the evaluation of the first, the participants worked with goals on a whiteboard in the patient's room. The data were collected in The Netherlands between February 2020 and June 2022. The data collection methods included the facilitator's logbook, observations, (group) interviews, charting activities and short surveys. Data analysis was conducted in weekly team sessions. The Guidelines for Best Practices in the Reporting of Participatory Action Research were followed.

**Results:**

In the first PAR cycle, the team learned that preparing an MTM with a patient did not enhance the patient's engagement in achieving their rehabilitation goals, but it was beneficial for the nurses' intermediate role between the patient and the multidisciplinary team. Clarity about responsibilities in the multidisciplinary team was a prerequisite for nurses to take on this role adequately. In the second PAR cycle, it became clear that working with a whiteboard in the patient's room had a positive effect on the patient's engagement in the rehabilitation process, and the nurses gained knowledge about a broader variety of professional rehabilitation domains.

**Conclusion:**

Through PAR, the nursing team learned two lessons: cooperating with patients through MTM preparation and working with whiteboards enhanced their patient‐centredness, but patients needed tangible goals to become engaged in their rehabilitation planning.

**Implications for the Profession and Patient Care:**

- Prepare the multidisciplinary team meeting with the patient, as discussing rehabilitation goals can indirectly boost motivation by making older patients feel seen and heard, even if they seem unable to fully participate in the conversation.
- Clarify responsibilities in the multidisciplinary geriatric rehabilitation team. This is a prerequisite for nurses to take on an advocacy role for patients in multidisciplinary team meetings.
- To enhance patient‐centred care, consider working with tangible goals on a whiteboard in the patient's room.

**Patient and Public Contribution:**

No public and patient involvement.

## 1 Introduction

Patient‐centredness is considered to be a core principle of the geriatric rehabilitation process. An important aim of geriatric rehabilitation facilities is to enable people to retain their independence for as long as possible and to maximise societal participation (Grund et al. 2020). To achieve this goal, services consider the patients' social circumstances as well as their functional abilities, viewing patients holistically rather than focusing solely on specific diagnoses (Grund et al. 2020; Kvæl et al. 2018). According to Kvæl et al. (2018), the main strategy to facilitate this holistic patient‐centred approach is to focus on patient involvement. Castro et al. (2016, 7) defined patient involvement as ‘a patient's rights and opportunities to influence and engage in the decision making about his care through a dialogue attuned to his preferences, potential and a combination of his experiential and the professional's expert knowledge’. In geriatric rehabilitation, a shared principle is to create a patient‐centred rehabilitation plan (Grund et al. 2020; Van Balen et al. 2019). Ideally, this plan is constructed by setting rehabilitation goals via a dialogue with each patient to ensure their preferences are assigned the appropriate priority (Grund et al. 2020; Levack et al. 2015). In addition, the creation of goals that hold personal significance—a process that involves individuals actively setting their own goals and communicating about these goals—has been linked to greater ownership of these goals, enhanced motivation to engage in subsequent rehabilitation activities and improved overall rehabilitation outcomes (Levack et al. 2015; Rosewilliam et al. 2016; Turner‐Stokes et al. 2015).

Patient‐centred work that involves rehabilitation goals is not self‐evident. Indeed, the authors have highlighted challenges related to practising patient‐centred goal setting, and poor patient engagement is often to blame. Smit et al. (2018) tested a new patient‐centred goal‐setting method for geriatric rehabilitation. The patients felt that the professionals largely dictated the steps towards discharge, and these steps were often unclear or not transparent to the patients. This view was confirmed in a longitudinal qualitative study by Lubbe et al. (2024). Moreover, research on geriatric rehabilitation and patient characteristics has revealed several aspects that might influence patient engagement. First, the average patient in this setting is portrayed at admission as being in a rather ‘fragile state’ due to their reason for rehabilitation. This state diminishes their ability to engage in goal discussions, making them less engaged in the process (Crawford et al. 2022; Tijsen et al. 2023a, 2023b). Van Balen et al. (2019) stated that delirium is one of the most important comorbid conditions in patients who are suitable for geriatric rehabilitation. Second, Rosewilliam et al. (2016) introduced other disempowering factors, including a lack of confidence because of not being ‘on home ground’ and fear of being branded a ‘troublesome patient’. Another challenge for shared goal setting is that it is usually new to patients in geriatric rehabilitation, making it difficult for them to understand what is expected of them (Crawford et al. 2022; Rose, Soundy, and Rosewilliam 2019). Patients do not expect to be active and to have a role in decision‐making (Rosewilliam et al. 2016), and they do not expect to perform daily activities themselves (Tijsen et al. 2023a). Lubbe et al. (2024) found that the level of involvement differs for each person: while some want a more active role, others prefer the professional to take the lead. Moreover, most patients gain an increasing grip on their situation during their rehabilitation process (Lubbe et al. 2024; Tijsen et al. 2023a, 2023b).

Based on earlier research, nurses can play a pivotal role in supporting patient involvement in the process of goal setting (Vaalburg et al. 2021). The primary aim of this support is to enhance the patient's understanding of their situation. Rose, Soundy, and Rosewilliam (2019) highlighted several supportive and educational tasks nurses can perform, such as clarifying the meaning of ‘goal’, outlining what to expect in goal‐setting meetings as well as the patient's potential role, helping patients divide long‐term goals into manageable steps, guiding patients in organising exercise schedules and summarising key points from discussions. Additionally, nurses apply skills from exercise sessions to everyday situations, making these sessions personally significant and thus also supporting active goal attainment activities (Kirkevold 2010). However, the increased acuity and complexity of patients admitted to inpatient rehabilitation units require nurses to focus more on general clinical care rather than rehabilitation care. This shift hinders a consistent, goal‐oriented approach to rehabilitation (Ehrlich et al. 2022). To support the above‐described role of a nurse, clear working methods that involve patients in their goal setting are necessary (Cameron et al. 2018; Loft et al. 2017).

On a geriatric rehabilitation ward in the Netherlands, a nursing team set out to improve their patient‐centred care by exploring how different working methods concerning goal setting and achievement can enhance patient involvement. They used participatory action research (PAR) for this endeavour. In our previous publication, we described this PAR process and evaluated the outcomes of involving nurses, as well as what hampered and facilitated collaboration between the nursing team and the researcher/facilitator (Vaalburg et al. 2024).

## 2 Background

The guiding principle in healthcare ‘quality thinking’ is to improve care based on reflecting and learning together with those involved. In PAR, participants and researchers undertake collective, self‐reflective inquiry to improve their practices (Baum, MacDougall, and Smith 2006). This iterative approach allows for the implementation of informed strategies, followed by further research to evaluate the outcomes. Empowering nursing professionals to actively engage in research fosters a culture of involvement and encourages them to voice and exchange their insights regarding effective practices. This collaborative approach can yield invaluable knowledge and expertise in navigating specific challenges within the field (Bekkema et al. 2021). We selected PAR over other more traditional qualitative methods (e.g., ethnography and interviews). Although those methods might also facilitate our understanding of the process of enhancing patient involvement through goal setting, they position researchers as external observers and do not involve people in the situation under study. Hence, using those methods would not enable the nursing team to refine their patient‐centred strategies, which was our aim.

This PAR was initiated by a nursing professor (R.J.J.G.) in collaboration with the manager of the geriatric rehabilitation unit at the nursing home. Their ambition was to enhance professionalisation of nursing care in geriatric rehabilitation. They assigned to the team the subject of improving their goal‐setting practice, considering this to be a key contributor to patient‐centred care. In the present paper, we describe the activities the nursing team carried out to explore the challenges concerning patient involvement through goal setting in geriatric rehabilitation using PAR.

## 3 The Study

The aim of this PAR was to provide insight into how a nursing team in geriatric rehabilitation can best promote patient‐centredness by involving patients, using goal‐setting activities as a means.

### 3.1 Method

#### 3.1.1 Design

The PAR was performed in two cycles, each consisting of five steps: orientation, diagnosis, development, action and evaluation. In the first cycle, a manual for preparing the multidisciplinary team meeting (MTM) with patients was designed and evaluated. In the second cycle, nurses and nursing students performed a pilot study on writing rehabilitation goals on a whiteboard in the patient's room. The findings are reported according to the recommendations of Smith, Rosenzweig, and Schmidt (2010; see Appendix S1). This paper describes the key elements of the project (e.g., the timeframe, the participants [and the nature of their roles], the process followed, the project outcomes and emergent actions, the timelines and tables to convey the project design), the experiences of the participants, and the challenges and limitations of this project.

#### 3.1.2 Study Setting and Period

This study was carried out on a geriatric rehabilitation ward (specialising in neurological and oncological rehabilitation) of a nursing home in the Netherlands. This ward had a capacity of 20 patients. Patients were admitted for post‐acute rehabilitation; most of them had pre‐existing functional deterioration or specific care needs. In the Netherlands, the average length of stay on a geriatric rehabilitation ward is 42 days (Vektis 2021). The nursing team consisted of an average of 18 nurses, of whom 12 actively participated in the PAR (see Table 1). At the start of the study, a Learning and Innovation Network (LIN) was installed on the ward: To improve the quality of care, three to four nursing students were added to the team for 20‐week internships. Nurses and nursing students worked together on practice‐based projects, constantly reflecting, learning and combining best practices, research evidence and patient perspectives (Albers et al. 2021). This PAR took place from February 2020 to June 2022.

**TABLE 1:** Nursing team members during the PAR.

Date	Team lead (RN)	Apprenticeship track students in training to become CNAs^a^	CNA^a^	RN^b^	Supernumerary bachelor's nursing students	Total Participating Nurses and Nursing Students
Period 1: 1 Feb. 2020	1	2	6	4	3	16
Period 2: 1 Sept. 2020	1	4	4	3	3	15
Period 3: 1 Feb. 2021	1	2	5	5	4	17
Period 4: 1 Sept. 2021	1	2	5	6	4	18
Period 5: 1 Feb. 2022	1	0	6	6	3	16

Abbreviations: CNA, certified nursing assistant; RN, registered nurse.

^a^ Compared to other countries, Dutch certified nursing assistant education is rather lengthy, consisting of a 3‐year practice‐oriented course (Van Wieringen et al. 2022).

^b^ Part of the team not mentioned in this table is a group of, on average, six RNs who mainly work evening, night and weekend shifts, bearing nursing responsibility for the entire nursing home. This group was not actively involved in the PAR.

#### 3.1.3 Researcher

A lecturer practitioner, a former registered nurse, facilitated the PAR process. She was present on the ward 1 day per week to support the nursing team in their action research activities, and she also provided educational sessions. The facilitator had a guiding role, aligning the team sessions as much as possible to the issues occurring in the work of the nurses, recapping the outcomes of the sessions and proposing the next steps in the process.

#### 3.1.4 Participants

There were four types of participants; in practice, the tasks of these different groups were intertwined. First, a core work group was established that consisted of a team lead and two senior team members of the nursing staff. This group made plans, monitored progress, discussed barriers and evaluated the project steps with the facilitator. If a team member was absent temporarily, then another team member would step into the core work group. The second group included the team members (also the core work group members) and the nursing students. The students were added to the team in five consecutive periods, with each group completing a 20‐week internship. In group sessions led by the facilitator every Wednesday over 2.5 years, the team members and students reflected on their practice, made plans for changing and improving practice, and performed practical research activities. During workdays, they tried out new practices. Some research activities were carried out mainly by the nursing students partly because they were doing internships, and partly because their bachelor's training made them more familiar with these types of activities. The third group comprised the (para)medical professionals working on the ward, including two physiotherapists, an occupational therapist and six consecutive physicians of which three were elderly care physicians and three were junior doctors. Although they had no formal role in this project, they joined some of the team sessions, when circumstances such as the coronavirus disease 2019 (COVID‐19) pandemic allowed, and provided feedback on the progress of some of the PAR steps. Finally, the fourth external project group consisted of academic experts, managers in geriatric rehabilitation and a patient representative. This group monitored the progress of the PAR on a bimonthly basis.

Patients on the ward were not involved in designing the PAR steps, so they cannot be labelled as PAR participants. Because of their relatively fragile condition, the choice was made not to burden them with these tasks. The regular turnover of the patient population also played a role in this decision, as well as the COVID‐19 pandemic that affected their health and the extent to which people could be together in one space. The patients did participate in the action and evaluation phases when new initiatives were tested and evaluated with them.

#### 3.1.5 Techniques, Procedures, Data Collection and Analysis

During this PAR, reflection, action and analysis were interconnected and occurred in a non‐linear manner. Because the challenge of PAR lies in understanding the subject under study by means of communication (Bergold and Thomas 2012), a core element was a weekly team meeting in which team members and nursing students discussed working on rehabilitation goals with the patients. Cook (2012) described this as ‘just talking’. The talking can lead to two important aspects of PAR: first, it is ‘fundamental to move beyond general conceptualizations of practice to deeper understandings’ (Cook 2012, 11). Second, talking—interaction in which there is trust that contributions will be valued and used appropriately—is key to knowledge development and change and, thus, to effective research (Wright et al. 2010). Bergold and Thomas (2012) labelled the above‐described method as a ‘focus group’, where participants are given the opportunity to discuss with each other in a safe setting and to deal with aspects of projects. It is a key instrument in creating a communicative space described by Habermas and is seen as a core principle in PAR (Dedding et al. 2022; International Collaboration for Participatory Health Research 2013).

Different approaches were used during the weekly sessions: in some sessions, theoretical models were used to initiate reflection, while in other sessions clinical cases were discussed and used to reflect on daily practice. In addition, various types of data were gathered prior, during and after the PAR process, and the results served as input for the sessions. The sessions were also used to share experiences and to reflect on new approaches that were tried. Team members, nursing students and the facilitator brought different knowledge and skills to the table. This approach facilitated the co‐construction of relevant solutions. Each session was verbally summarised at the end, and the facilitator took notes. Analysing and validating findings was done in a communicative way to ‘enhance ownership’ (Bergold and Thomas 2012). The preliminary conclusions drawn from former sessions were summarised, adding new knowledge and experiences—and thus also triangulating—to continuously deepen, refine and get to the core in an iterative process. The findings were distributed to the team members (whether or not they attended the sessions) in a special newsletter, through flip‐over sheets hung on the team room wall, in personal emails and in student presentations. The facilitator monitored the process in a logbook. Work group members constantly corrected or confirmed the accuracy of the interpretations by, for example, giving feedback on reports written for member checks, and they were also accountable to the external project group and management. Table 2 provides an overview of the data collected in this study.

**TABLE 2:** The two participatory action research cycles.

Cycle and phase	Objective	Data collection	Analysis	Documentation (form and by whom)
*Cycle 1: preparation of the multidisciplinary team meeting with the patient*				
Orientation and diagnosis: February 2020–June 2020	To explore (how) do we as a nursing team support patient‐centredness through goal setting, and whether there aspects of our work that need to be improved	Method: weekly group sessions with team members and students about their patient‐centred goal‐setting practice Data: facilitator's notes; flip charts of discussions in sessions	Analysis (summarising preliminary conclusions of previous sessions, adding new knowledge and new experiences—thus also triangulating—to continuously deepen, refine and get to the core in an iterative process) in weekly group sessions with team members and students	Problem and solution catalogue of aspects that could be improved, prepared by students and facilitator PowerPoint presentation by students of findings Flip chart produced by the facilitator with a summary of orientation and diagnosis phase to inform the entire team Report by the facilitator documenting data collection, analysis and outcomes
	To explore whether patients have goals, know their goals and work on their goals	Method: 12 interviews with patients by bachelor nursing students regarding rehabilitation goals Data: students' notes of answers on the printed interview protocol		
Development: September 2020–January 2021	To describe the current practice in preparing the MTM in order to develop an MTM path	Method: student nurses interviewed team members about their current practice in preparing MTM Data: students' notes Method: student nurses observe team members preparing MTM Data: students' notes	Analysis (summarising preliminary conclusions of previous sessions, adding new knowledge and new experiences—thus also triangulating—to continuously deepen, refine and get to the core in an iterative process) in weekly group sessions with team members and students	Students' report of interviews, observations and questionnaire findings Poster of the seven‐step plan about how to prepare an MTM created by the students Presentation of the seven‐step plan about how to prepare an MTM by the students Two newsletters with instructions about how to work with the seven‐step plan Assignment about preparing an MTM for next group of students by the facilitator and core work group
	To learn about patients knowledge about and experiences with MTMs	Method: questionnaire administered to 13 patients by students about patients' knowledge about and experiences with MTMs Data: students' notes		
Action: February 2021–June 2021	To try out the seven‐step plan	Nurses and students started working with the seven‐step plan		
	To involve as many team members as possible in applying the seven‐step plan To reflect on added worth of patient involvement	Method: larger group session with team members (24 March) on patient involvement through preparation of MTM Data: flip charts and facilitator's notes	Analysis (summarising preliminary conclusions of previous sessions, adding new knowledge and new experiences—thus also triangulating—to continuously deepen, refine and get to the core in an iterative process) in weekly group sessions with team members and students	
Evaluation: February 2021–June 2021	To evaluate experiences with the seven‐step plan with core work group members	Method: three interviews with core work group members by the facilitator about testing the seven‐step plan Data: interview transcripts	Analysis (summarising preliminary conclusions of previous sessions, adding new knowledge and new experiences—thus also triangulating—to continuously deepen, refine and get to the core in an iterative process) in weekly group sessions with team members and students Counting the number of preparations in electronic patient records Counting the number of times the patient was reported to be involved based on the electronic patient records	Bar chart PowerPoint by the facilitator Report by the facilitator, nurse and students Report by the facilitator Instruction on how to prepare MTM
	To provide a quantitative and tangible overview of progress in the use of the seven‐step plan	Method: over 12 weeks, team members tracked whether the MTMs were being prepared and, if so, whether the patients were involved Data: quantitative overview		
	To reflect collaboratively on new practice of using seven‐step plan	Method: preparatory MTM talks with patients by nurses/students were observed by facilitator and subsequently evaluated with nurses/students Data: facilitator's notes		
	To evaluate the preparatory talks with patients	Method: interviews with eight patients by facilitator about their experiences with MTM preparation Data: facilitator's notes of answers on the printed interview protocol; facilitator's notes		
	To evaluate new practice of using seven‐step plan with nurses and other members of the multidisciplinary team	Method: group interview about new practice of how nurses prepare MTMs with core work group members, physiotherapist and physician by facilitator Data: flip charts and transcription		
		Method: survey among team members about their role in MTMs Data: digital file with answers		
*Cycle 2: involving patients in their rehabilitation process using whiteboards*				
Orientation and diagnosis: September 2021–January 2022	To answer the following question: how can the MTM on our ward ensure better patient involvement during the rehabilitation process?	Method: four patient interviews by a bachelor's student about involvement in their rehabilitation process Data: interview transcripts	Analysis of interviews by the bachelor's student	Bachelor's thesis PowerPoint presentation by the student
Development: February 2022–June 2022	To discuss and plan whiteboard project approach	Method: core work group session about the whiteboard project approach Data: transcription		Project plan for whiteboard project
	To asses patients' knowledge about their exercise goals	Method: survey/measurement 1 among all 20 patients on the ward Data: survey results	Analysis (summarising preliminary conclusions of previous sessions, adding new knowledge and new experiences—thus also triangulating—to continuously deepen, refine and get to the core in an iterative process) in weekly group sessions with team members and students	Students' report Instruction written by students on how to fill in the chosen whiteboard setup
	To choose a white board setup	Method: two possible whiteboard setups were presented Data: the team members indicated their preference on a shared form	Counting preferences	
Action: February 2022–June 2022	To try out the process of negotiating about goals and writing them on the whiteboards in patients' rooms	Method: experimenting with writing goals on the whiteboards in patients' rooms with eight patients Data: notes from students and team members		
Evaluation: February 2022–June 2022	To learn about the added value of writing goals on the whiteboards in patients' rooms	Method: weekly team sessions with team members and students (focus groups) about their experiences in working with goals on the whiteboard in the patients' room Data: facilitator's notes; flip charts of discussions in sessions; pictures of whiteboards	Analysis (summarising preliminary conclusions of previous sessions, adding new knowledge and new experiences—thus also triangulating—to continuously deepen, refine and get to the core in an iterative process) in weekly group sessions with team members and students	Students' reports PowerPoint by students
	Second inventory of patients' knowledge about their exercise goals	Method: survey/ measurement 2: all 20 patients on the ward were asked whether they knew their exercise goals Data: survey results		

Abbreviation: MTM, multidisciplinary team meeting.

#### 3.1.6 Rigour

The rigour of this PAR was ensured based on its dependability, credibility and transferability (Stahl and King 2020). The weekly sessions created a continuous process of knowledge development in which member checking was a standard procedure, and this endeavour enhanced credibility. Extra member checking was done by presenting summary reports to the project group and checking the assumptions with the core work group. Additionally, semi‐structured interviews, observations by students and the facilitator, short surveys and charting actions used in the PAR contributed to ‘method triangulation’. The facilitator and first author performed this PAR project under supervision of four tutors. To ensure dependability, peer debriefing and peer review were integral parts of their meetings. In a safe environment, the facilitator was invited to employ a self‐critical attitude, considering how her opinions affected the research. Transferability was guaranteed by documenting the PAR process in detail; this endeavour included keeping records of discussions and notes, on any decisions made during the PAR.

#### 3.1.7 Ethical Considerations

The study was approved by the Medical Ethics Review Committee of VUmc supervising this PhD project (decision number 2019.400). This committee concluded that the Medical Research Involving Human Subjects Act (WMO) did not apply to this study (Central Committee on Research Involving Human Subjects 2024). Participation was voluntary, and the work and personal boundaries of the nurses and students were considered (e.g., meetings were established at times convenient to care needs patient care). Informed consent was obtained from the students and nurses before commencing the data‐collection activities that we audio recorded. Following Articles 20, 21 and 22 of the Declaration of Helsinki (World Medical Association 2024) about the patient's right to self‐determination and the right to make informed decisions regarding participation in research, the patients were informed, asked for consent and given the opportunity to decline participation. Consent was recorded. Note that there are two types of consent: written and verbal. The patients were asked to provide written consent when they completed a survey (e.g., before and after the whiteboard project) or were interviewed (e.g., the interview to evaluate the MTM preparation). The patients were also informed that no personal information would be captured, and that if their experiences were to be reflected in research reports, they would be presented in a generalised form. The patients were asked to provide verbal consent when the team tried out actions. We made this choice for verbal consent because the interventions were within the scope of normal care, without risk and we did not collect personal data.

## 4 Findings: A Description of the Two PAR Cycles

### 4.1 Cycle 1: Preparation of the MTM With the Patient

#### 4.1.1 Orientation and Diagnosis: Suboptimal Continuity of Care

Four months were devoted to creating a safe space for exchanging experiences and knowledge about the core subject of the PAR: *(How) do we as a nursing team support patient‐centredness through goal setting, and are there any aspects of our work in this field that need improving?* The result was a problem and solution ‘catalogue’, listing barriers and facilitators in this process of working with rehabilitation goals to enhance a patient‐centred process. The team found that a fair share of the patients on their ward were not actively engaged in their rehabilitation process. The patients assumed that they ‘were rehabilitated’. The team realised that some preconditions for working with goals were not met, which negatively affected patient engagement in the rehabilitation process. First, one of the problems in the catalogue was the patients' limited perception of what rehabilitation entails and what is expected from them in it:

In today's session we discussed the fact that patients in hospital are told: ‘You'll be going to the nursing home for a short time in order to gain strength before going home.’ The patients think that ‘gaining strength’ means resting. So, we discussed exactly what we should say to patients to explain what revalidation involves. (Facilitator's logbook. Cycle 1, phase: orientation)

Another item in the problem catalogue was a lack of continuity of care. The team stated that a more consistent approach by all nurses towards patients concerning their rehabilitation goals was needed. This would enhance clarity for the patients and support patient engagement.

Subsequently, the problem and solution catalogue was presented by the nursing students in a special meeting with as many team members present as possible. By applying stickers to their preferred solution, the team chose to focus on the two preconditions for working with goals. Through better preparation of the MTM with the patient, in which they would discuss progress on rehabilitation goals, they aimed to better inform and involve patients in their rehabilitation process. Simultaneously, updating the goals in the patient record was intended to enhance continuity of care. Consequently, all involved professionals would adopt a consistent approach, providing patients with greater clarity about the rehabilitation process.

#### 4.1.2 Development: Whose Continuity Is Paramount?

The next group of nursing students set out to develop a working procedure for the preparation of the multidisciplinary care plan meeting with the patient. They interviewed team members about their current practice. For example:

Student nurse: Why do you consider it important to prepare the MTM with the patient? Nurse: By sitting down together, you are more informed about the patient's treatment plan. (Student report, by student nurses 2, 8, and 9, on preparing the MTM. Autumn 2020)

They also observed colleagues, studied the literature about patient involvement in MTMs, and interviewed patients about their experiences. In team sessions, theory about and practice of patient involvement were discussed. One of the findings described in the students' report included:

It was notable that involving the patient in the preparation of an MTM is not mentioned right away. When questioned further, several answers emerged: one person indicated that you can observe a lot during ADLs [activities of daily living] and discuss this with the patient. You then ask the patient how they experience this, taking into account the goals being worked on. You observe this well. Another nurse said that they print out the rehabilitation plan and take it to the patient […] to evaluate how much progress there is in achieving the goals. (Student report, by student nurses 2, 8, and 9, on preparing the MTM. Autumn 2020)

Based on the interviews, the observations and the literature, the students constructed a seven‐step plan on how to best prepare the MTM with the patient (see Table 3).

**TABLE 3:** Seven‐step plan for preparing the multidisciplinary team meeting.

Seven‐step plan for preparing the multidisciplinary team meeting		
1	Check which patients will be discussed in the next MTM	Preparation by the nurse
2	Study a number of reports of the patient, so as to form an idea of the patient's progress	
3	Update the progress on rehabilitation goals if necessary	
4	Write a summary in the electronic patient record on the progress on each goal	
5	If possible, provide care to the patient shortly before the MTM, in order to make your report more accurate	Preparation with the patient
6	Inform the patient about the upcoming MTM, ask for their opinion on their progress: do they have any additions to your input? Ask about: Activities of daily living, pain, mobility, mood	
7	Incorporate the patient's information in your report	

#### 4.1.3 Action: The Type of Questions Must Be Tailored to the Patient's Situation

A seven‐step plan was presented to the team, posters showing the steps were hung up in the ward, and in several sessions, the steps were introduced to and discussed with the team members. Everyone started working with the seven‐step plan. A new group of nursing students arrived on the ward, and the team members introduced them to the new procedure.

#### 4.1.4 Evaluation

Two criteria were identified prior to the evaluation. First, the MTM should be prepared with the patient according to the seven‐step plan. Second, in the electronic patient record a report should be written clarifying how the patient felt about their progress. For 12 weeks, the nurses tracked whether the MTMs were prepared and if they were prepared with the patients. More than half (55%) of the MTMs were prepared by a nurse per the preparatory report (*n* = 78). Somewhat less than a quarter (22%) were prepared with the patient. Three reasons were given for not involving the patient in the preparation: cognitive impairment, language barrier and time. In addition to the quantitative tracking, several other methods were applied to evaluate the preparation of the MTM with the patient (see Table 2).

Several lessons were learned. These lessons could be grouped under three themes: patients' experiences with MTM preparation, preparing the MTM provides useful information about the patient's process, and lack of clarity about responsibilities among nurses.

##### 4.1.4.1 Patients' Experiences With MTM Preparation: The Added Value of MTM Preparation Was Not Always Clear

With eight patients, the preparatory talks were evaluated in the same week. The talks did not seem to result in the patients gaining a comprehensive understanding of their rehabilitation trajectory. The nursing team had expected this understanding to enhance patient involvement. Four patients did not quite remember the talks. Four patients experienced the preparatory talks positively as a form of attention or it boosted their trust in the team of clinicians to know that they were concerned with their progress:

We [*facilitator and student nurse 1, author AMV*] realised that although not all patients are able to oversee the whole trajectory, they should nevertheless be informed about what is discussed in the multidisciplinary team meeting. We approached a female patient to inform her of the multidisciplinary team's concerns. She was relieved to hear that these were the same worries she had herself. (Facilitator's logbook. Cycle 1, phase: evaluation)

The patients were not fully aware of the main purpose of the interviews, specifically to hear their views on their rehabilitation progress so that their concerns could be addressed by the nurse in the MTM, thus promoting a patient‐centred rehabilitation process. According to the nurses, there were several reasons for this lack of awareness. First, not all patients were familiar with this active role, due to their previous hospital stay, in which they were not expected or able to play an active role. Second, some were cognitively not able to understand rehabilitation as a process due to tiredness or the impact of a disease. Finally, in the nurse–patient dynamics, the patients seemed more inclined to help the nurse gather their information than putting forward their own specific worries.

Even though it became clear that the conversation did not obviously contribute to enhanced patient involvement, the nurses wanted to proceed with working via the seven‐step plan. They emphasised the indisputable importance of talking with the patient about steps to be taken towards discharge:

Student nurse 2 described a patient who reacted negatively to the preparatory meeting. He indicated that he thought it was ‘useless’ and that he had already discussed everything with the doctor. […] Afterwards it turned out that he was very concerned about the functioning of his pacemaker and had no room in his head to focus on (other aspects of) his rehabilitation. What we took away from this as an important lesson is that a ‘negative experience’ with a patient when it comes to preparing the MTM should not stop you from persevering. It may well be that the patient also has to grow into this way of working—to get used to it. (Facilitator's logbook. Cycle 1, phase: action)

##### 4.1.4.2 Preparing the MTM Provides Nurses With Useful Information About Each Patient's Process

During this PAR, the nurses experienced the added value of MTM preparation with the patient. First, it allowed them to optimally represent the patient in the meeting:

Nurse 2 said about preparing the MTM (my representation of her words): Previously in the MTM I could only properly determine the state of affairs for my own patients. I tried quickly to determine the status of the other patients from the reports, but I was hoping, rather than knowing, that I was doing it right (makes a doubtful face). Now, with my colleague's preparation, I am much more confident. I know someone took a good look at it. I can rely on my colleague's reporting. (Facilitator's logbook. Cycle 1, phase: action)

Second, because time was spent with the patient to more thoroughly discuss progress, certain issues were raised that otherwise would not have come up:

Student nurse 6 delves deeper into how to build up intake through the mouth. She read in the patient's record that the speech therapist gave permission to introduce soft foods gradually. She asks why the patient is taking only the nutritional drink. The patient replies that she tried mashed potatoes, but they gave her terrible stomach problems; now the patient wonders how she should approach the introduction of solid food. […] Student nurse 6 asks if the speech therapist gave her any tips. […] The patient then indicates that it is not so much a speech therapy problem but more of a dietary problem; swallowing is fine, but the stomach has become unaccustomed to solid food. The result of this conversation is that student nurse 6 consults the dietitian again. (Facilitator's logbook. Cycle 2, phase: action)

In general, non‐nurse workers noticed a greater and more valuable contribution in the MTM from the attending nurses.

#### 4.1.5 Lack of Clarity About Responsibilities Among Nurses

During this PAR, it also became clear that the nurses felt a certain apprehensiveness in trusting their assessment of the patients' situations. For example, they hesitated putting a patient on the consultation agenda as well as adding patient goals to or removing redundant goals from patients' records. They left this task to the physician. The nurses had to be convinced that their attention was expected to help make progress on all the goals, not only on the nursing goals focused on activities of daily living; therefore, the nursing team should be aware of goals the physiotherapist and other health professionals work on with the patient. The same applied to mental aspects of rehabilitation: in their preparatory talk with the patient, experienced nurses also focused on the mental aspects of the rehabilitation process such as motivation and cognition. When these aspects were missing in the plan, some of the nurses did not address them. The important role of the electronic patient record as a supporting tool in the process of preparing the MTM became clear.

### 4.2 Cycle 2: Involving Patients in the Rehabilitation Process Using Whiteboards

#### 4.2.1 Orientation: Use of Whiteboards Might Enhance Patient Involvement

After the first cycle, the team concluded that the preparatory MTM talks had not resulted in the patient being more engaged in their rehabilitation process. As a next step, the core work group asked one of the students on the ward to focus on the following question for her bachelor's thesis: *how can the multidisciplinary team on our ward ensure better patient involvement in achieving their rehabilitation goals?* Based on interviews and a brief literature review, her recommendations were: the use of whiteboards in the patients' room for writing on small exercise goals; working with smaller attainable goals instead of end goals; a weekly evaluation talk; and working with a primary nurse approach, assigning nurses to specific patients. The core work group chose the use of whiteboards in patient rooms to set small goals and to evaluate regularly as the next step in this PAR aimed at improving patient involvement through goal setting.

The nurses and students, inspired by a research article by Revello and Fields (2015) on working with goals on whiteboards, together surveyed the patients regarding whether they could articulate their exercise goals, with whom they exercised, and if the whiteboard was already in use for the purpose of goals. Additionally, they paid a visit to a neighbouring ward to learn about their practices with whiteboards.

#### 4.2.2 Diagnosis: Most Patients Were Aware of Their Exercise Goals

The survey showed that most patients on the ward could describe their exercise goals. However, the team still wanted to experiment with writing these goals on the whiteboards. They believed this approach could strengthen the nursing team's role in supporting patients to actively engage in their rehabilitation process and exercise.

#### 4.2.3 Development and Action: Whiteboard Information Had to Be Presented Clearly

First, the team brainstormed to determine which headings to put on the whiteboard; examples were sought from neighbouring wards, and patients were invited to chip in with their preferred headings. Two possible whiteboard setups were developed, and all team members were invited to voice their opinion. Eventually, one was chosen, mainly because of its clarity.

Next, exercise goals were written on a whiteboard in the patient's room. Flyers were put up in the room to inform all colleagues. The inclusion criteria for the patients were they were able to understand Dutch and motivated to participate, and the nurse saw opportunities for progress. This email from one of the nurses describes her attempt to set up goals together with the patient:

I asked the patient how he thought his rehabilitation was going. […] His answer: I can move a bit more than I could. When I then asked what else he would like, his answer came quickly and clearly: I want to be able to walk. I asked him: […] What do you need to be able to do this? He said that he felt ‘bad’ that he could not stand on the bridge. He said he had no strength. I asked: Where is there no strength—arms or legs? It took a while for him to answer, but he realized it was his arms. I explained that I wanted to write a small goal on the whiteboard in his room and that his goal for the day would be to practice. Then I gave him weights to practice with. He seemed to agree with this and be happy with it. (Email from nurse 2, January 2022)

#### 4.2.4 Evaluation

The whiteboard pilot study was carried out with nine patients. In the weekly meetings, the nurses and nursing students shared their experiences. There were two guiding questions: *how did you collaborate with the patient in establishing the goals on the whiteboard, and were other healthcare professionals involved? How did the patients experience working with the whiteboard goals?* Table 4 gives an overview of the goals on the whiteboards and the lessons learned.

**TABLE 4:** Whiteboard goals and lessons learned.

Reason for patient's admission	Goal(s) on whiteboard	How did the goal come about?	What lessons did we learn?	Quotes for illustration
Patient first in maintenance phase during chemotherapy, then in rehabilitation phase	Write down what I ate Write down the questions I have, as well as the answers! Today I am going to make my bed and join the others for one meal in the dining room Tidy the desk	The goals related to the “chemo brain” came about largely through conversations between the patient and nurse Goals relating to energy management were established during discussions between the patient and nurse after the nurse had consulted the occupational therapist	A whiteboard can be useful in cases of forgetfulness resulting from a “chemo brain” Looking for a connection with personal goals can help a patient remember goals The patient is afraid of being overloaded; the goals on the board should not give her the feeling that she has to do a lot Limited exercise tolerance (energy management) is a good topic for the whiteboard. However, the nursing staffs' knowledge is not specific enough to discuss this with the patient and set appropriate goals—consultation with an occupational therapist is thus necessary Goals can work as a lower limit. The patient does more than is stated on the board, trying to do two rounds more than the five on the board	*After every chemo treatment I'm in a haze, so I write down as much as possible* (Patient's quote in student nurse report) Observation: *I noticed that the patient was very motivated to do more once she had reached her goal of five laps. She probably thought that an extra lap or two wouldn't hurt since the first five went easily. However, she did not take into account that her current condition does not allow her walk 10 laps without a walker. She overlooked this fact, or simply did not think about it. Afterwards she said herself that it was too much, but that she really wanted to prove herself* (Student nurse report)
Patient in oncological treatment process	Climb the stairs every day	The goal was drawn up with a physiotherapist	This patient is well aware that she has to climb stairs every day, but she appreciates the attention and encouragement involved in filling out and adjusting the whiteboard together	
Patient rehabilitating after new hip and stroke	Advice: You may put your full weight on your leg You may bend your leg at least 90° You may walk around in your room with one elbow crutch *Things to practice* Right hand: pay attention to how you hold it! 5 min of ball squeezing—after breakfast, after lunch, after dinner	The physiotherapist had given exercise instructions, and the nurse wrote these on the whiteboard in simple language	The patient likes structure in the day. The assignments on the whiteboard provide this The patient needs clarity. Numerical instructions appeal to him The whiteboard was too full for the patient and the language unclear. The text was shortened and the language simplified	*This gentleman has a great need for structure and clarity. For that reason, we considerably reduced the text on the whiteboard and made certain things more concrete for him* (Facilitator's logbook, Cycle 2, phase: action)
Patient in post‐urosepsis rehabilitation	A timetable lists the times the patient must empty his bladder by opening the valve of the catheter	The goal came about because the patient wanted more independence. He wanted to get rid of the catheter. The doctor suggested working with a valve. The nurse came up with an interim solution: The patient was allowed to empty the bladder himself via the valve	The patient finds it annoying to be constantly reminded of things due to social anxiety. He prefers to have as few contact moments as possible He likes the timetable on the board and follows it—it gives him independence The patient is not ashamed of the goals on the board	*I drew up the goals with a nursing colleague. In the protocol on using the catheter valve we saw that the bladder had to be emptied every 3 to 4 h. […] With this information, my colleague and I filled in the board. The gentleman was happy that this information was on the board and was motivated to achieve this goal because he wanted to be free from his catheter as soon as possible* (Student nurse report)
Patient rehabilitating after stroke and wrist fracture	On Thursday you will take a shower	After having discussed the need for personal hygiene, the patient agreed to have a shower the next day. Because of his ambivalence regarding water and soap, the nurse wrote the agreement to shower on the whiteboard	The agreement on the whiteboard works like a “big stick”, (it's actually going to happen!). The whiteboard makes the agreement concrete, and the patient has all day to get used to the idea	
Patient rehabilitating after total hip replacement, with the residual symptoms of a stroke	I am going to work with clay to improve mobility in my hands At 19:00 h I will cycle to improve mobility in my arms and legs	The patient received assignments from the physiotherapist and wanted these to be put on the whiteboard	The patient sticks to the agreements that have been made because he needs structure in his day The patient needs challenging goals. For him, goals should be framed in maximum terms	*If it says 3 min, I won't do a minute more* (Patient interview)
Patient rehabilitating after cellulitis and possible stroke	Squeeze pillow with my hand Walk 2–3 m using walker for transfer Get changed while in bed	The goals were established during a conversation between the patient and a physiotherapist	The patient made a lot of progress with rehabilitation, but progress has slowed down now. It could be that updating the goal on the board more often would improve motivation Goals must be meaningful for the patient Daily reports are provided on how the patient's transfers are progressing. The functioning of the hand is less well reported Patient is not ashamed of goals on the board	*I need to learn how to tie knots. But I don't have a boat—why do I have to learn how to tie knots? I would like to go to the market independently again, have drinks with my friends. I would like to walk again, get in the car or a bus* (Patient interview) *Being changed in bed—I don't have a problem with this being on the whiteboard; I'm not ashamed of it. But I can also just let the nurses know myself, when it is needed* (Patient interview)
Patient rehabilitating after a cerebral infarction	Practice pronouncing sounds twice a day Rest for 60 min after lunch	The patient received assignments from the speech therapist The patient has a regular habit of resting after lunch when she is at home. To remind the nurse of this, she would like it written on the whiteboard	Talking about the goals and giving compliments about them helps the patient continue practising It was educational for the nurses to discuss with the patient what they would put on the whiteboard—they discovered that the patient has speech therapy exercises and also what problems she experiences. Nursing can now report on this better The whiteboard is informative for the daughters	*The patient herself sees progress in pronouncing sounds. She likes it when she receives compliments and her efforts are recognised. This makes her want to practice more frequently* (Student nurse report) *I have reported on this goal several times. The patient always practices on the days that I am there. However, I do not always see my colleagues' evaluations of this goal, so I do not have insight into whether she actually practices every day* (Student nurse report)
Patient rehabilitating after left cerebral infarction	Rest 60 min after an activity like therapy	The patient's family indicated that she is tired, and the patient confirmed this. The physiotherapist and nurse agreed for her to take rest periods throughout the day	In addition to the rest goal, the nurse formulated a goal about paying attention to the left arm. The patient thought this was too much for the whiteboard and gave priority to the fatigue. The board should not become too full. We learned from this that the responsibility for paying attention to this point lies more with the nursing staff; also, that the whiteboard can be an instrument for shifting more control to the patient The patient forgot about the whiteboard and still needs to be reminded orally to rest. The rest goal on the whiteboard serves as a reminder for colleagues	*We (student nurse 4, student nurse 7, nurse 4) discussed a patient about whom student nurse 7 had filled the whiteboard. This patient has two issues: a lack of energy/fatigue and the attention paid to her left arm. We looked at how the whiteboard corresponds with the rehabilitation plan in the electronic patient record (ECD); the whiteboard is not intended to become an additional ‘plan’/instrument, alongside the ECD. It was a very good search that we carried out together. Everyone studies the electric patient record, and we looked at where and how we could include the energy issue in her rehabilitation plan. We decided it could be included as an evaluation with the mobility goal and also in the report as an important observation (fatigue after therapy)* (Facilitator's logbook, Cycle 2, phase: action) *The whiteboard is of no use to the patient. I don't see any progress towards the goal. The patient only rests when the nurse tells her to* (Student nurse report)

##### 4.2.4.1 Whiteboard Goal Setting Contributed Positively to the Rehabilitation Process

In eight of the nine patients, working with the whiteboard goals somehow made a positive contribution to their involvement in their rehabilitation process. About half of the goals on the whiteboard came about through a prescription from another healthcare provider (i.e., physiotherapist and speech therapist); the other half of the goals came about through nurse–patient collaboration, sometimes requiring a consultation with another healthcare provider. A form of patient involvement could also be seen in the case of prescribed goals. In one case, the discussion about which goals should be written on the whiteboard revealed an important insight. The patient prioritised the goal she felt capable of working on independently and asked the nursing team to take responsibility for the other goal. The lesson here was that the whiteboard could function as an instrument to literally give and take responsibility. There were several other lessons the nursing team learned: the whiteboard lent itself to all kinds of topics, not only physical exercises; clarity was important—the whiteboard should not contain too many messages (too many goals could overwhelm and prevent the patient from taking action) and no jargon; a connection between exercise goals and the patient's personal life was desirable; and patients did not seem to mind goals about personal hygiene written on the board.

##### 4.2.4.2 The Impact of Goals on the Whiteboard on Patients' Behaviour Differed

The nursing team also learned lessons about the actual impact of the goals on the whiteboard on patients' behaviour. Setting goals on the whiteboard influenced their involvement in their rehabilitation process in different ways. For three patients, the goals on the whiteboard provided them with the structure they needed during the day; it helped them gain independence. Personal character influenced the way goals on the whiteboard were interpreted: as an ultimate goal or as a minimum level to reach; the personal attention that came with talking about their whiteboard goals with the nurse could enhance motivation, even though some patients knew pretty well what to exercise; and when goals were adjusted less often because progress stabilised, motivation could be affected in a negative way. Finally, the whiteboard also served to inform family.

##### 4.2.4.3 Whiteboard Goal Setting Led to Nurses Gaining Broader Rehabilitation Expertise

Finally, a lesson was learned about continuity of care in the multidisciplinary team. Up until this project, the nurses mainly reported on progress in activities of daily living and mobility. Other issues were primarily the concern of the therapist, and the patient and received less attention from the nurse. However, the whiteboard helped the nurse to play a broader role and to integrate these goals into their work:

Speech therapy exercises and moments for rest are posted on the whiteboard. […] It was very useful for student nurse 7 to discuss with her what they would put on the whiteboard because the student nurse now knows that she has speech therapy exercises, and that she has problems with them. Student nurse 7 is now able to report on this better. (Report of evaluation interview with patient in room 17, and with student nurse 7)

After the pilot study with the nine patients, an instruction was written, and the team started working according to this instruction.

## 5 Discussion

The main aim of this study was to provide insights into how, through goal‐setting activities, a nursing team in geriatric rehabilitation could refine their patient‐centred strategies. Using PAR, the team tried out and reflected on two working methods on goal setting and achievement. Using these methods, they hoped to enhance engagement in the rehabilitation process, first through talking about goal progress during MTM preparation, and second through shared decision‐making on planning smaller daily goals.

### 5.1 Lessons About Enhancing Patient Involvement Through Goal Setting

Regarding patient involvement, by discussing goals, two lessons were learned. The first was that although patients valued the MTM preparation positively, these talks did not specifically provide the patient with a comprehensive understanding of their rehabilitation trajectory and thus increase their involvement in achieving their rehabilitation goals. The patient appeared to be a vulnerable person not quite able to participate optimally in talks about the rehabilitation process and its associated goals. Based on this finding, we contemplated the following question: should we stop preparing the MTM with patients? Kristjánsson and Thórarinsdóttir (2024) warned against ‘forced participation’ dynamics in their study on constrained engagement of vulnerable adults. They labelled patient participation not based on patients' wishes to participate as ‘bad’ participation; for example, patients can feel stressed or fatigued and thus not be able to participate. We chose not to stop. Although the patient's vulnerability stood in the way of equal communication about goals, discussion of rehabilitation progress with the patients positively impacted their motivation by making them feel seen and heard. It seems that the patients considered ‘being listened to’ and ‘having reciprocal communication’ as crucial for their involvement, a view consistent with the findings reported by Kvæl, Bergland, and Eldh (2023), who pilot tested the 12‐item Patient Preference for Patient Participation scale with older patients in intermediate care. Still, to encourage patient involvement in rehabilitation goals, the team experimented with using whiteboards in patient rooms to set and evaluate small goals.

The second lesson the nurses learned was that collaboration on the setting of tangible whiteboard goals can make a positive contribution to the patient's involvement in their goals. Strikingly, this element aimed at supporting goal achievement has not been adequately considered in rehabilitation goal‐setting interventions (Kang et al. 2022). Hence, this way of supporting patients in working on their rehabilitation goals requires more attention. Consistently, Wattel et al. (2023) designed, evaluated and tested a practical guideline on goal setting that emphasised the central role of goal talk throughout the rehabilitation process, as this gives the patient control over their own rehabilitation.

The nurses found that the impact of the whiteboard goals on the patients' behaviour differed substantially. The experiences during the PAR revealed diverse patient identities (see Table 4, column 4), and each of these identities had different implications for the collaboration with the patient on the setting of whiteboard goals. This did not seem solely related to the phase of their rehabilitation: as suggested by Tijsen et al. (2023a, 2023b), it also depended on the patient's personality. While the whiteboard goals provided some patients with structure and reminders, others mainly appreciated the attention and the motivational power that came with the collaborative goal setting. Some patients gained independence in following their exercise programme, whereas others indicated they still needed the oral encouragement of the nurses.

In summary, while conventional empowerment models in healthcare consider patient involvement to be a central and self‐evident condition, we learned that these models can overlook the complexity of patient involvement in practice. Involvement is not a ‘one‐size‐fits‐all’ concept. Rather, it is highly individual and varies according to each person's health, worries, context, values and character, among other characteristics. Because of these personal differences, the primary role of the healthcare professional is to be responsive to the patient's needs, values and preferences with respect to the content of encounters, the style of communication and involvement in decision‐making (Pluut 2016). In a previous study, this delicate process of estimating the amount of control a patient is able to wield in the goal‐setting process and tuning in on that level of control came about as an important aspect of geriatric rehabilitation nursing (Vaalburg et al. 2023).

### 5.2 Lessons About the Role of the Geriatric Rehabilitation Nurse

The PAR approach provided room for reflection and shared learning. Knowledge about enhancing patient participation was not the only outcome from this PAR. Our findings are similar to those reported by Steensgaard et al. (2021): nurses began noticing unclear or inefficient work procedures and learned several lessons about the role of geriatric rehabilitation nurse. First, preparing the MTM can provide nurses with useful information about a patient's progress. Kočo et al. (2022) confirmed the positive effect of an improved preparation of cases on the quality of discussions and the decision‐making process. It strengthens the nurse in their role as patient advocate during the MTM; the preparation helped nurses better understand the patients' aspirations (and their barriers) and thus enhanced patient‐centred care. The second lesson was that a barrier to optimally fulfil this advocacy role was the lack of clarity among nurses about responsibilities. Doornebosch, Achterberg, and Smaling (2024) studied factors that influence interprofessional collaboration in geriatric rehabilitation and found that implicit working processes and unclear policies regarding working methods hindered collaboration and reaching joint objectives.

The third, unexpected, lesson came from working with the whiteboards. The nurses realised that not all goals were previously shared with the multidisciplinary rehabilitation team—those goals were mainly been focused on activities of daily living and mobility. The extra communication via the whiteboard goals informed the nurses about broader aspects of rehabilitation, such as the management of energy, speech and nutrition. Thus, working with the whiteboards helped the nurses gain knowledge that spanned a variety of professional domains, facilitating interprofessional collaboration and enhancing the quality of care (Sinclair, Lingard, and Mohabeer 2009). For the nurse, having this broader knowledge can be seen as a prerequisite for their intermediate role in the rehabilitation team.

### 5.3 Strengths and Limitations

A specific feature of rehabilitation is the multidisciplinary composition of the team. In this PAR, we did not formally include non‐nursing members, and this can be seen as a limitation. This was a deliberate choice because the nursing profession in geriatric rehabilitation in the Netherlands still requires professionalisation (Vaalburg et al. 2023). Thus, we focused solely on nurses to give as many nursing team members as possible the ability to participate, and to share and develop their knowledge. Our decision not to include family was practical: involving family would have increased the scope of the research, requiring more time, resources and logistical planning. Nevertheless, their role in geriatric rehabilitation is important, and future research should preferably include them.

Conducting PAR on a single ward may limit the generalisability of the findings to other settings, as the characteristics of the ward—in our case, the neurological and oncological patient population—might not reflect those of other wards or institutions. However, in the second cycle of the PAR we found that the goals the patients would like to work on were quite diverse in nature (energy management, speech, mobility, continence, etc.) and served a broad range of purposes (independence, structure, motivation, etc.). Hence, we assume that these results apply to a broader group of geriatric rehabilitation patients.

Another limitation is that the facilitator experienced a few dilemmas regarding the opportunities available for nursing team members to participate in the PAR. Due to the lack of time earmarked for the nurses to participate in actual research activities, the students in collaboration with the facilitator mainly executed these tasks. Spalding (2009) argued that this ‘imbalance’ can also be positive: it is a sign of respect for the limited time of the nurses, which leads to more of a coordinator role for the facilitator. However, we presume that a job description allowing nurses to focus on quality improvement, along with an organisational power structure that connects quality management to improvement projects on wards, would generally lead to more sustainable results. Steensgaard et al. (2021) mentioned that similar organisational obstacles influenced the potential of their improved approach concerning goal setting.

Another limitation was the turnover of nursing staff and students due to school schedules, private reasons, as well as the COVID‐19 pandemic. This turnover led to discontinuity because ambitious personnel left the word to work in COVID‐19 cohorts. Therefore, it was necessary for the facilitator and the members of the core work group to perform extra activities to keep everyone involved. The work group members acted as ambassadors by discussing the PAR steps with their colleagues during daily work activities. To ensure everyone stayed informed, flip charts summarising individual sessions or specific parts of the PAR process were displayed on the team room wall. At one midterm evaluation, the work group signalled that not every team member was properly engaged. A special newsletter was started for this reason. Back‐office information showed that the newsletter was well read.

The facilitator's more dominant role also had the risk of influencing the PAR; due to her background, as a former nurse she saw a more central position of nurses in the multidisciplinary team as a means to enhance patient‐centredness through goal‐setting activities. By constantly checking her assumptions with the work group and the team, the facilitator tried to minimise this effect. Moreover, her own experiences and predispositions influenced the process because she did not have a note‐taker and, in her central position, she constantly translated team members' issues, stories and experiences regarding patient‐centred rehabilitation.

Finally, the facilitator experienced a lack of influence due to her external position vis‐à‐vis the nursing team. This was mainly reflected in keeping agreements on carrying out certain actions, as patient care always takes priority. There were two strategies applied to overcome this barrier: first, to increase the chances of successful uptake of the interventions, she tried to steer the team towards changes that aligned as much as possible with the team members' existing routines. This included contacting the quality officer to find out about organisational policies and adhering to them. Second, to keep the team members involved, she adapted to their fast pace, although this sometimes meant letting go of the researcher's perspective of a thorough research procedure.

The most important strength of this PAR was the significant amount of time spent on learning and doing by the nurses and the nursing students. There was a sufficient amount of time and number of opportunities for the nurses and students to share their knowledge based on their practical experiences, which contributed to the fun and success of this PAR, and the LIN was crucial to this outcome. Several creative methods were applied during the team sessions to disclose tacit knowledge and to support the nurses and students to explicitly share their experiences. Another strength was our focus on practical implementation and evaluation of interventions aimed at improving patient involvement in achieving their rehabilitation goals. By trying out and evaluating working methods (e.g., whiteboards for goal setting) and involving the patient in the MTM preparation, we gathered valuable insights into the feasibility of these methods. This emphasis on real‐world application ensured relevance for clinical practice.

## 6 Conclusion

This PAR provided insights into how a nursing team through goal‐setting interventions can enhance a patient‐centred rehabilitation process in which older patients are actively involved. Preparing the MTM with the patient did not lead to the patient developing a comprehensive understanding of their rehabilitation trajectory, nor did it increase their involvement in achieving their rehabilitation goals. However, it did help the nurses understand how the patients experienced their progress and thus enabled the nurses to play an advocacy role in the MTMs. Clarity about responsibilities in the multidisciplinary collaboration, a central feature of geriatric rehabilitation, was found to be a prerequisite for nurses to take on this role adequately. Additionally, the nursing team experienced that working with a more tangible goal‐setting instrument like a whiteboard in a patient's room aligned more with patient's abilities and could have a positive effect on an older patient's involvement in the process of rehabilitation. The patients' personal ways of working with the whiteboards gave the nurses deeper insights into the patients' preferences regarding the content of the encounters, the patients' style of communication and the patients' involvement in the rehabilitation process. Overall, working with a whiteboard and letting patients put all kinds of goals on the board allowed the nurses to gain knowledge about a broader variety of professional domains. This helped them to further develop as geriatric rehabilitation nurses and will also contribute to the development of the entire profession in that field.

## Author Contributions

All authors agreed to the final version of this manuscript and met at least one of the following criteria: (1) substantial contributions to conception and design, acquisition of data or analysis, and interpretation of data; (2) drafting the article or revising it critically for important intellectual content.

## Ethics Statement

The Medical Ethics Review Committee of VUmc concluded that the Medical Research Involving Human Subjects Act (WMO) did not apply to this study (2019.400) (Central Committee on Research Involving Human Subjects 2024).

## Conflicts of Interest

The authors declare no conflicts of interest.

## Peer Review

The peer review history for this article is available at https://www.webofscience.com/api/gateway/wos/peer‐review/10.1111/jan.16774.

## Supporting information


Appendix S1.

## Data Availability

Data available on request due to privacy/ethical restrictions.
