# Supplementary material for: Participatory Action Research to Enhance Patient‐Centred Goal Setting in Geriatric Rehabilitation: A Nursing Team's Quest
Source: J Adv Nurs. 2025 Jan 24;81(10):6646–64. doi: 10.1111/jan.16774 (PMC12460944; doi:10.1111/jan.16774)
Supplement: Supplementary file 1 — Appendix S1. [file JAN-81-6646-s001.docx]

**Appendix 1**
Guidelines for Best Practices in the Reporting of Participatory Action Research (PAR) according to Smith et al. (2010).

| Guideline | Section we reported in |
| --- | --- |
| Plan Ahead for Organizational Structure  Adapt conventional organizational headings,  or Consider deriving organizational structure from project design elements or emergent themes,  or Consider a chronological or narrative framework | A chronological narrative framework was used in section *Findings*. |
| Convey the Key Elements of the Project | |
| How was the project initiated? | See *Design* section:  This PAR was initiated by the nursing professor (XX) in collaboration with the manager of the geriatric rehabilitation unit at the nursing home. They assigned to the team the subject of improving their goal setting practice, considering this a key contributor to patient-centred care. |
| What was the project’s timeframe? | Described in *Study setting and period* This PAR took place between February 2020 and June 2022. |
| Who were the participants and/or co-researchers? | Described in subparagraph *Researcher* and subparagraph *Participants*. |
| What was the extent of their participation and the nature of their roles? | Described in subparagraph *Researcher* and subparagraph *Participants*. |
| What was the process within and/or the methodology of the project? | See section *Techniques, procedures, data collection, and analysis*.  See *Table 2*. |
| What were the project outcomes and/or emergent actions? | This is described in *Findings.* The first cycle of action did not enhance patient participation in their rehabilitation process. Therefor the team chose a second action. This did have a positive effect on patient participation in their rehabilitation process. Additionally the team learned lessons about refining their patient centered strategies. |
| What comes next (if the project is ongoing)? | Our manuscript does not report beyond the timeframe of this PAR. |
| Consider charts, timelines, tables, or other graphics to convey part or all of the project design. | See Table 2. |
| Convey the Experiences of Co-Researchers | |
| Pay attention to who is writing the article and how their voices and experiences are represented. | In the *Discussion* section we reflect on the more dominant role of the facilitator. |
| Pay attention to who is not writing the article and how their voices and experiences are represented | Several citations reflect the experiences of the nurses and nursing students. See section *Findings*. |
| What were the personal outcomes of the project? | See section Discussion: Work group members acted as ambassadors by discussing the PAR steps with their colleagues during daily work activities. See *Discussion*.  Lot’s of time for learning and reflection which was appreciated and which contributed to the fun.  Inefficient work procedures were noted.  Nurses experienced the positive effect of MTM preparation. |
| Address the Challenges, Pitfalls, and Limitations of the Project | |
| What were they? | See *Strengths and limitations* section:  1.Lack of earmarked time for the nurses for actual research activities.  2. The turnover of staff and students in the nursing team, e.g. due to school schedules, private reasons, and also the covid-pandemic caused discontinuity because ambitious personal left the word to work in Covid-cohorts.  3. The facilitator’s more dominant role also held in it the risk of influencing the PAR; due to her background, as a former nurse she saw a more central position of nurses in the multidisciplinary team as a means to enhance patient-centered goal setting. |
| How were they managed? | See *Strengths and limitations* section:   1. The students in collaboration with the facilitator mainly executed these. 2. The facilitator and the members of the core workgroup to employed extra activities to keep everyone involved. 3. By constantly checking assumptions with the work group and the team, the facilitator tried to minimize this effect. |
| What can we learn? | See *Discussion* section:   1. We presume that a job description that allows for nurses to work on quality improvement, as well as an organizational power structure that connects quality management with improvement projects on wards, in general would lead to more sustainable results. 2. Constant communication about activities and outcomes is important to keep a whole team involved. |
